# Glycosylation of Dentin Matrix Protein 1 is critical for osteogenesis

**DOI:** 10.1038/srep17518

**Published:** 2015-12-04

**Authors:** Yao Sun, Yuteng Weng, Chenyang Zhang, Yi Liu, Chen Kang, Zhongshuang Liu, Bo Jing, Qi Zhang, Zuolin Wang

**Affiliations:** 1 Tongji University, School of Stomatology, Laboratory of oral biomedical science and translational medicine, Shanghai, China, 200072; 2 Harbin Medical University, Institute of Hard Tissue Development and Regeneration, Harbin, China, 150086; 3 Tongji University, School of medicine, stem cell center, Shanghai, China, 200072

## Abstract

Proteoglycans play important roles in regulating osteogenesis. Dentin matrix protein 1 (DMP1) is a highly expressed bone extracellular matrix protein that regulates both bone development and phosphate metabolism. After glycosylation, an N-terminal fragment of DMP1 protein was identified as a new proteoglycan (DMP1-PG) in bone matrix. *In vitro* investigations showed that Ser^89^ is the key glycosylation site in mouse DMP1. However, the specific role of DMP1 glycosylation is still not understood. In this study, a mutant DMP1 mouse model was developed in which the glycosylation site S^89^ was substituted with G^89^ (S89G-DMP1). The glycosylation level of DMP1 was down-regulated in the bone matrix of S89G-DMP1 mice. Compared with wild type mice, the long bones of S89G-DMP1 mice showed developmental changes, including the speed of bone remodeling and mineralization, the morphology and activities of osteocytes, and activities of both osteoblasts and osteoclasts. These findings indicate that glycosylation of DMP1 is a key posttranslational modification process during development and that DMP1-PG functions as an indispensable proteoglycan in osteogenesis.

Proteoglycans (PGs) are high molecular weight molecules that are composed of GAG chains covalently linked to a core protein. In extracellular matrix (ECM), proteoglycans account for less than 10% by weight of all extracellular matrix proteins. However, they play key roles in tissue development that include filling the extracellular matrix space, maintaining the shape of organs and providing structural strength, assembling porous hydrated gels that enable the matrix to withstand compressive forces, regulating activities of secreted proteins and mediating interactions between cells and the ECM^1^. Furthermore, proteoglycans from bone matrix are indispensable in osteogenesis. Their specific roles include regulating cell proliferation; controlling extracellular matrix formation, mineral deposition, and bone remodeling; and maintaining the mechanical properties of bone^2^. Loss of specific proteoglycans could lead to skeletal defects during bone formation, mineralization and remodeling. Well-known proteoglycans in bone tissue include Biglycan and Decorin^2^,^3^. Biglycan (*Bgn*) is a type of proteoglycan that is highly expressed in bone, and *Bgn*-deficient mice exhibit impaired postnatal bone formation and early onset of osteoporosis^4^.

Dentin matrix protein 1 (DMP1) is a major extracellular matrix protein and plays a key role in osteogenesis. In *Dmp1*-null mice, morphological changes in bone and bone mineral loss are obvious^5^. The osteomalacia and rickets phenotypes could be attributed to inability of osteocytes to mature and reduced levels of serum phosphate that occurs when *Dmp1* gene is removed^5^. Previous protein chemistry studies showed that DMP1 undergoes several posttranslational modification steps, including proteolytic processing and phosphorylation^6^. After proteolytic processing, DMP1 is presented as N- and C-terminal fragments in bone matrix^7^. Highly phosphorylated DMP1 C-terminal fragments accelerated the mineralization of bone, and re-expression of the C-terminal fragment of DMP1 fully reversed bone rickets in *Dmp1*-null mice^8^. The C-terminal protein is recognized as the functional fragment of the DMP1 protein; however, the biological function of the DMP1 N-terminal fragment is still unknown.

In bone matrix, the N-terminal fragment of DMP1 is present in two forms: one is the 37 kDa core protein, and the other is a high molecular weight proteoglycan that contains a glycosaminoglycan chain (named DMP1-PG)^9^. The GAG chain is linked to Ser^74^ in rat DMP1 (Ser^89^ in mouse DMP1)^9^, which is a highly conserved glycosylation site. In long bones of mice, DMP1-PG is mainly expressed in osteoid and articular cartilage^10^,^11^,^12^. Studies also have shown that an S89G substitution (S^89^-G^89^) could block the glycosylation of DMP1 *in vitro*, which confirmed that Ser^89^ is the GAG chain attachment site in mouse DMP1^13^. In this study, to determine the role of DMP1 glycosylation in osteogenesis, substitution of the DMP1 glycosylation site S^89^ with G^89^ was performed in mice using the knock-in method. Therefore, this mouse model contains only mutant S89G-DMP1 and not endogenous DMP1. The expression of DMP1-PG is dramatically decreased in the bone matrix of S89G-DMP1 mice. Bone phenotypes in these mice include bone mass, bone mineralization, trabecular bone thickness and number, and cortical bone thickness and stiffness. Furthermore, the osteocyte lacuna diminished in size, and the expression levels of many bone formation markers were down-regulated. Based on these findings, we speculated that DMP1-PG could function as a typical bone matrix proteoglycan and regulate osteogenesis.

## Results

### Substitution of the DMP1 glycosylation site S^89^ with G^89^

**Figure 1:**
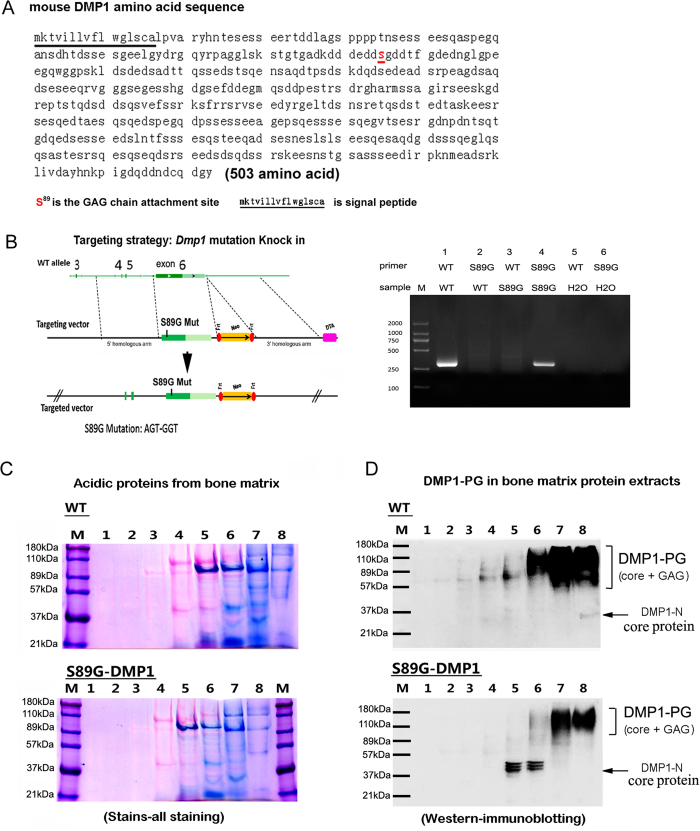
Substitution of amino acid for DMP1 glycosylation site. (**A**) Amino acid sequence of DMP1, and the GAG chain attachment site of DMP1; (**B**) Mutation strategy for DMP1 glycosylation site and genotyping result; (**C**) Stains-All staining for acidic proteins extracted from long bone of WT and S89G-DMP1 mice; (**D**) Detection of DMP1-PG by Western immunoblotting. Compared with WT mice, expression of DMP1-PG protein was significantly decreased in long bone of S89G-DMP1 mouse.

Initially, the S^89^-G^89^ mutation (S89G-DMP1) in mice was confirmed by genotyping (sup-Fig. 1). Acidic proteins including DMP1 were extracted from long bones and applied to a Q-Sepharose column to purify the DMP1 components. Using a gradient of NaCl (0.1 to 0.8 M) containing 6 M Urea, eluted components was collected in fractions #1–8. DMP1 protein extracted from S89G-DMP1 mice was compared with extracts from WT mice. These samples were subjected to Stains-All staining, and the expression profiles of acidic proteins extracted from long bone matrix are shown in Fig. 1C. By Western immunoblotting, DMP1-PG was detected in fractions #6–8 (elution buffer was 0.6 M–0.8 M NaCl in 6 M Urea) in both WT and S89G-DMP1 groups. DMP1-PG appeared as a smear between the 89 kDa and 180 kDa markers. After S89G mutation: the DMP1-PG smear was shorter and weaker in S89G-DMP1 mice group; more DMP1-N core protein bands could be detected in the fractions of S89G-DMP1 mice group (Fig. 1D). These results show that DMP1 glycosylation could be effectively inhibited by a point substitution at glycosylation site S^89^-G^89^.

### Bone Morphological changes in S89G-DMP1 mice

**Figure 2:**
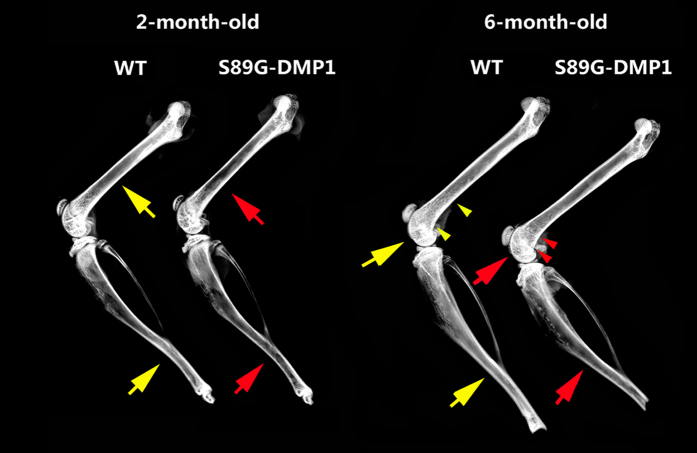
Comparison of the X-ray images between WT and S89G-DMP1 long bone. In both 2- and 6-month-old mouse, the bone density of S89G-DMP1 is lower than WT, and the cortical bone of WT mouse (yellow arrows) is wider than S89G-DMP1 mouse (red arrows). The trabecular bone under the growth plate in S89G-DMP1 mouse (between red arrowheads) was shorter in than WT mouse (between yellow arrowheads).

**Figure 3:**
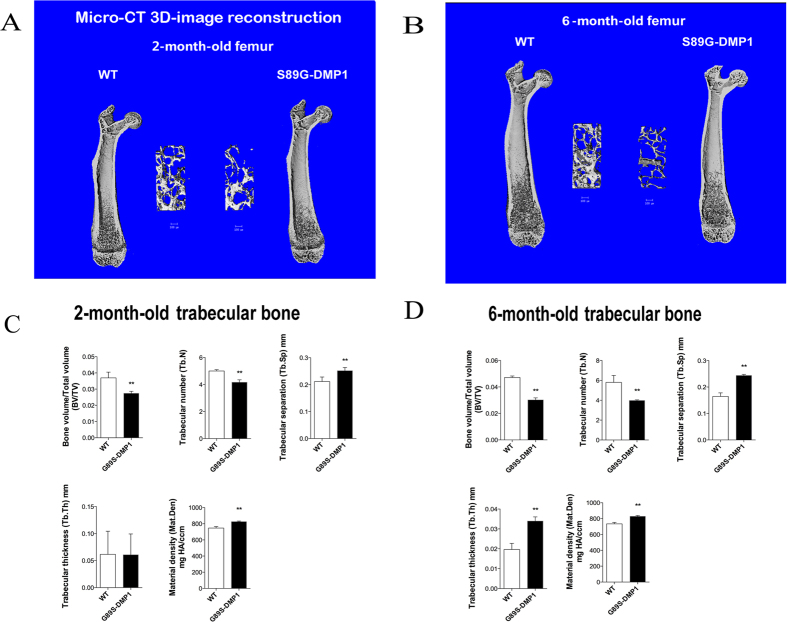
Micro-CT analyses for femurs. (**A,B**) The micro-CT reconstruction images of long bone and trabecular bone under growth plate for bone 2- and 6-month-old mouse. (**C**,**D**) The bone density, trabecular number, trabecular thickness in S89G-DMP1 mouse is lower than WT mouse. But the Mat-Den of S89G-DMP1 bone is slight higher than WT mouse.

**Figure 4:**
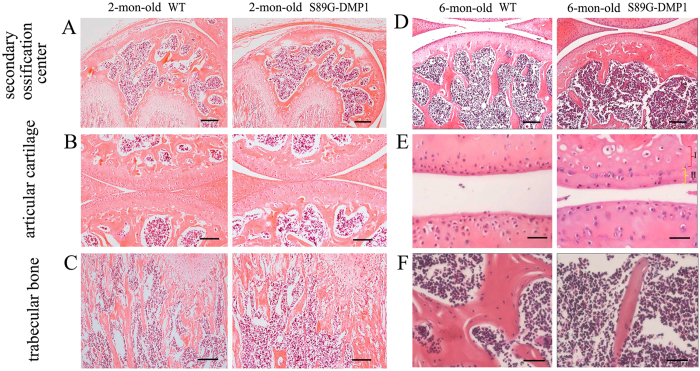
H&E staining for long bone of 2- and 6-mon-old WT and S89G-DMP1 mouse. Compared with WT mouse: bone loss could be detected in the secondary ossification center (**A**,**D**); the articular cartilage is thinner (**B,E**); the trabecular bone in the S89G-DMP1 mouse is less than WT (**C**,**F**). In 4A, Bars = 400 μm; In (**B–D**), Bars = 200 μm; In (**E–F**), Bars = 100 μm.

X-ray images indicated that the bone density of S89G-DMP1 mice was lower than that of WT mice at both 2 and 6 months old (Fig. 2). In addition, a decrease in both cortical bone thickness and the length of trabecular bone was observed (distance between two arrowheads). Trabecular bone loss in DMP1-S89G mice was detected by μ-CT analysis (Fig. 3A,B). Compared with WT mice, the bone volume/total volume ratio (BV/TV), trabecular number (Tb.N) and trabecular thickness (Tb.Th) all decreased, while the trabecular separation (Tb.Sp) and the material density (Mat.Den) increased in S89G-DMP1 mice. The differences between these two groups were more obvious in 6-month-old bones (Fig. 3C,D). There was obvious trabecular bone loss in both the secondary ossification center and the metaphysis area (Fig. 4A,B). There were also changes in chondrocyte number and morphology in articular cartilage of 6-month-old S89G-DMP1 mice at (Fig. 4D,E). The tidemark (between the red and yellow areas) indicated that the mineralization of articular cartilage could be accelerated by reduced expression of DMP1-PG.

### Morphological and functional changes in osteocytes

**Figure 5:**
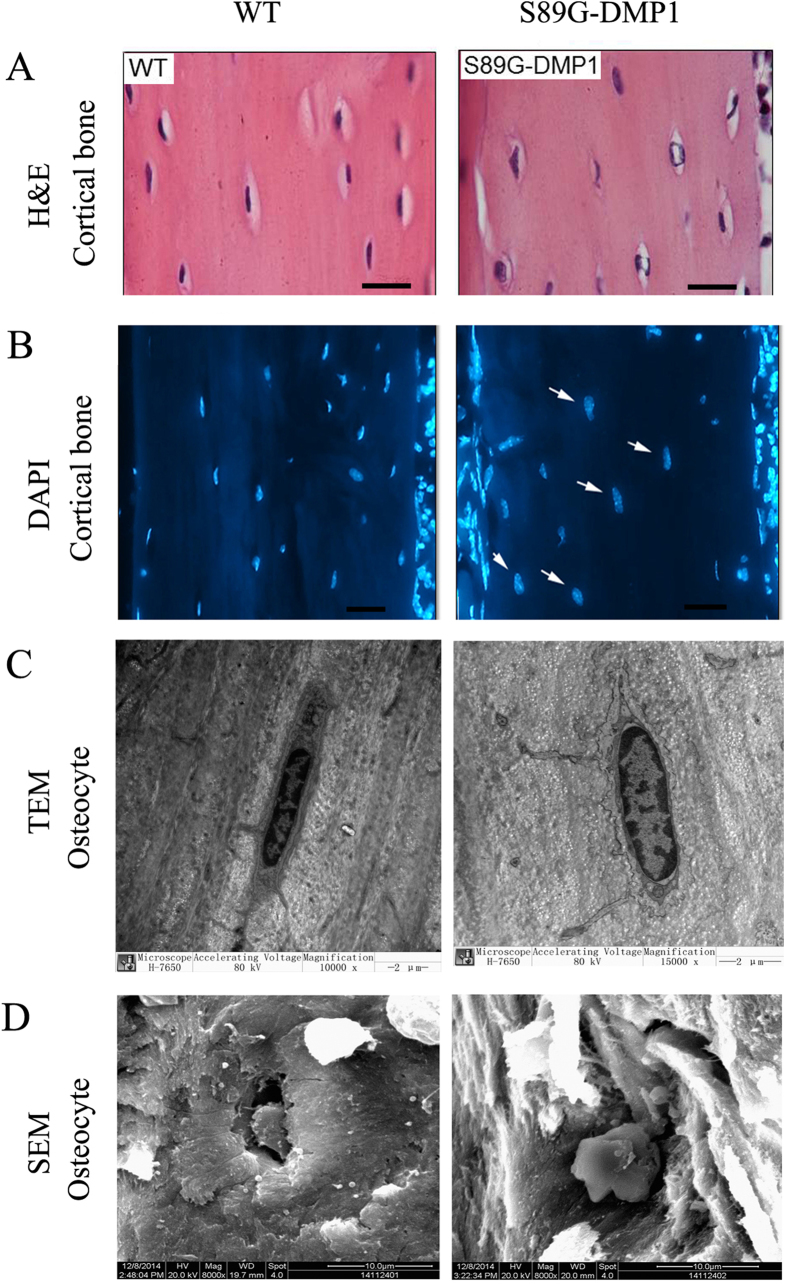
Cells changes in cortical bone. (**A**) Cell lacunas were obviously narrow in S89G-DMP1 cortical bone, Bars = 30μm; (**B**) Osteocyte nucleus of S89G-DMP1 were bigger than WT mouse in cortical bone, Bars = 30 μm; (**C**) Increasing of the nuclear-cytoplasmic ratio of S89G-DMP1 mouse by TEM; (**D**) Differences morphological characters of bone lacunas and osteocytes between WT and S89G-DMP1 mouse by SEM: the cell lacuna shrinked, and more mineral could be observed surrounding the osteocyte.

**Figure 6:**
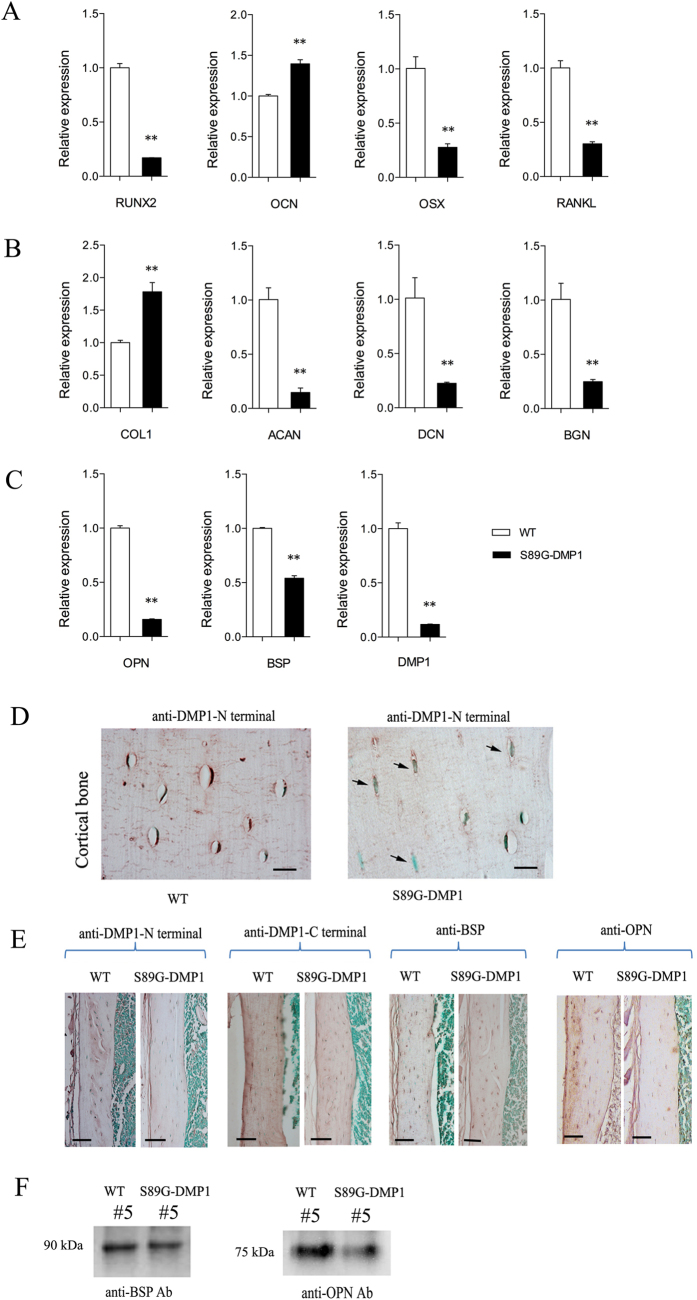
Expression levels of genes and proteins changes in bone of S89G-DMP1 mouse. (**A**) RT-QPCR quantitation of bone formation and resorption genes from long bone; (**B**) RT-QPCR quantitation of bone matrix proteins from long bone; (**C**) RT-QPCR quantitation of SIBLING proteins from long bone; (**D**) By using anti-DMP1-N terminal mono 9b6.3 antibody, DMP1-PG expression were detected surrounding most of the osteocytes in WT, but signals lost in S89G-DMP1 osteocytes which the cell lacuna surrounding it shrinked (arrow heads), Bars = 20 μm; (**E**) Expression of matrix SIBLING protein family (BSP, OPN, DMP1) in cortical bone of S89G-DMP1 mouse was tested by IHC, the expression level of acidic bone matrix proteins were lower than WT mouse, Bars = 100 μm; (**F**) The protein expression levels of both BSP and OPN were down regulated in bone matrix of S89G-DMP1 mouse.

The long bones of S89G-DMP1 mice show shrinkage of the osteocyte lacuna. H&E staining shows that part of the cell lacuna is thinner those of WT mice (Fig. 5A). A number of nucleuses of osteocytes were larger in S89G-DMP1 mice, which was confirmed by DAPI staining (Fig. 5B). In addition, the nucleocytoplasmic ratio was increased by using TEM (Fig. 5C). In SEM images, after the loss of the PG form of DMP1, the 3D shape of osteocytes and the cell lacuna changed (Fig. 5D). In S89G-DMP1 mice, the RNA expression of bone formation markers (Runx2, Osx), bone mineralization markers (DMP1, OPN, BSP), a bone resorption marker (Rankl) and proteoglycans (Acan, Dcn, Bgn) were down-regulated (Fig. 6A–C). Bone matrix proteins, including DMP1, BSP and OPN, were down-regulated in DMP1-S89G mice (Fig. 6D,E). It is worth noting that in the cortical bone of the S89G-DMP1 mouse, the shape of osteocytes changed (indicated by arrows in Fig. 6D), and the expression of the DMP1-N terminal was much lower than in the WT mice (Fig. 6D). In addition, Western immunoblotting confirmed the down-regulation of BSP and OPN protein expression (Fig. 6F).

### Activity changes in osteoblasts and osteoclasts

**Figure 7:**
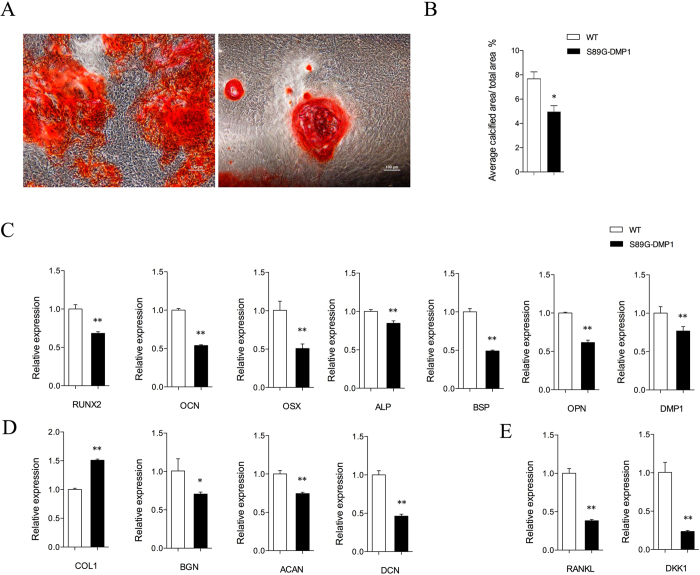
Activities of osteoblasts. (**A**) Comparation of calcified nodules secreted by osteoblasts from WT and S89G-DMP1 group by Alizarin red staining; (**B**) The quantitation of calcified nodules; (**C**) The bone formation genes expressed by osteoblasts; (**D**) Collagen I and bone proteoglycans genes expressed by osteoblasts; (**E**) Bone resorption genes expressed by osteoblasts.

**Figure 8:**
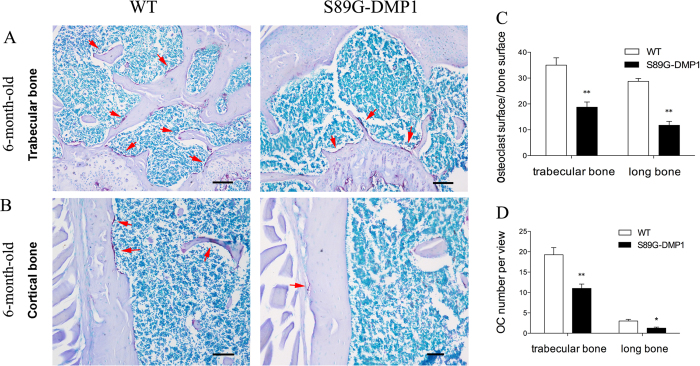
Number changes of Osteoclasts. Osteoclasts number was compared in trabecular bone area (**A,C**) or cortical bone area (**B,D**) in 6-month-old WT and S89G-DMP1 mouse. Bars = 100 μm.

Alizarin Red staining showed that the osteogenic differentiation of osteoblasts was delayed after the GAG chains of DMP1 were partially removed. The markers for monitoring bone formation and bone resorption were both down-regulated. Furthermore, three major bone matrix proteoglycans were also down-regulated after the loss of DMP1-PG (Fig. 7). There were fewer osteoclasts in the cortical and trabecular bones of S89G-DMP1 mice than in those of WT mice (Fig. 8).

### Biomechanical testing of femur and tibia

**Figure 9:**
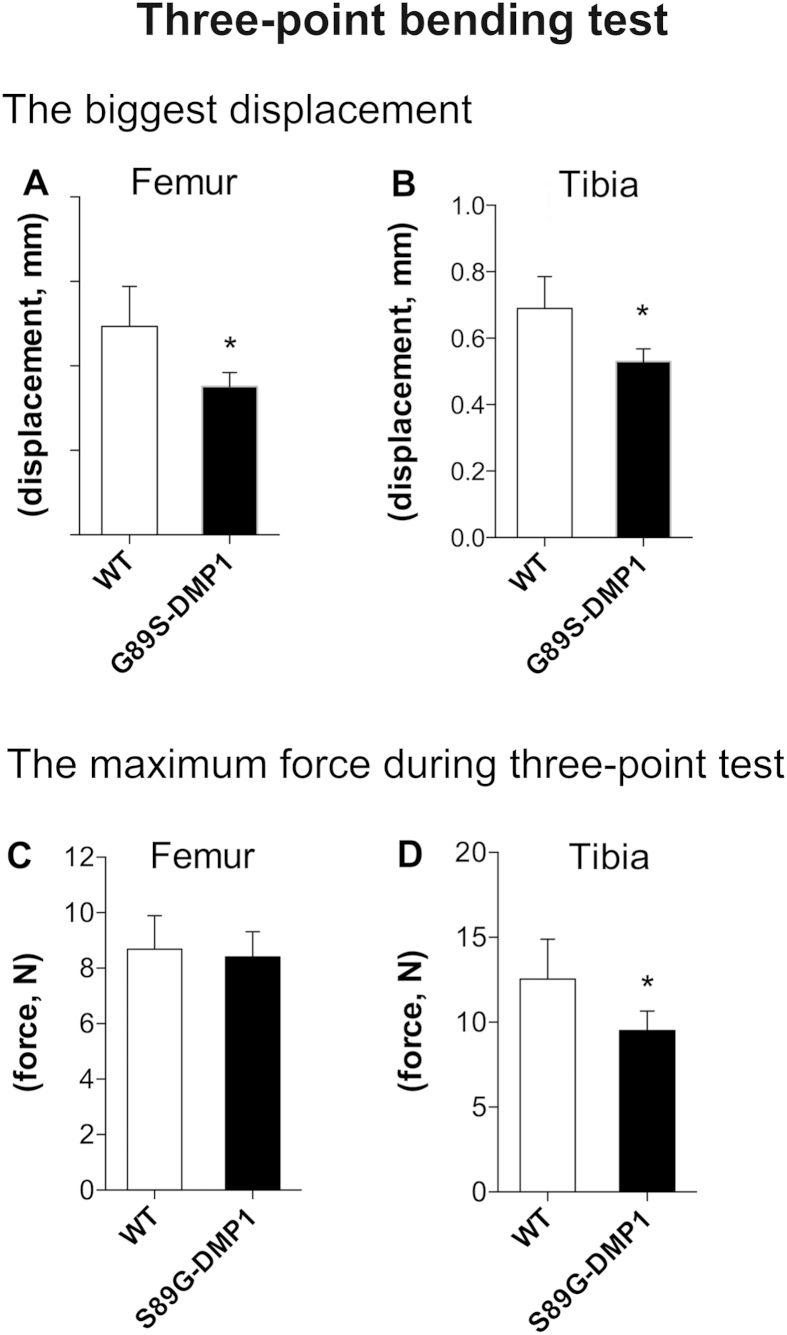
Three points bending test for femurs and tibias of S89G-DMP1 mouse. Comparation of the flexural capacity of femurs (**A,C**) and tibias (**B,D**) between WT and S89G-DMP1 mouse.

The mechanical strength of the femoral diaphysis was tested. The bending strength of the femoral and tibia diaphysis in 2- and 6-month-old mice was significantly different between the two groups of mice. The three point bending experiments showed that, after the DMP1-S89G mutation, the bending resistance ability of long bone also decreased (Fig. 9).

### Osteogenesis-related genes/pathways involved after the mutation of DMP1

**Figure 10:**
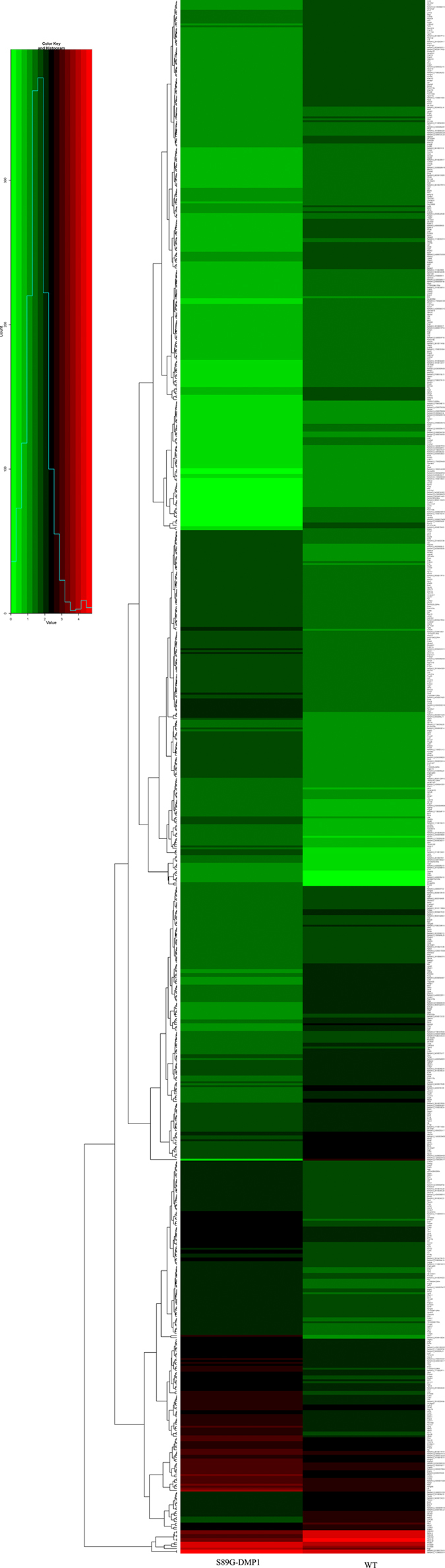
Heat Map analyses of Differential expressed mRNA. Differential expressed mRNA sequencing results were compared between WT and S89G-DMP1 mouse long bone.

Differential gene expression between the two groups was evaluated by RNA sequencing. Compared to WT mice, 901 transcripts were up- or down-regulated in the long bones of S89G-DMP1 mice: 444 genes were down-regulated and 457 genes were up-regulated (Fig. 10). According to the Kyoto Encyclopedia of Genes and Genomes (KEGG), these changes in gene expression can be categorized into 22 pathways (Table 1). Among these pathways, both ECM-receptor interactions and TGF-β pathways are important for bone and cartilage formation. In the ECM-receptor interaction pathway, Col1, Col2, Col3, Col5, Col6, Col11, SPP1, Ibsp, Sdc3, Tnc and Thbs4 were up/down-regulated. In the TGF-β pathway, E2f4, Acvrl1, Dcn, Id2, Id3, Mapk3, Ppp2cb and Thbs4 were up/down-regulated. Furthermore, molecules in the cell adhesion pathway, the tight junction pathway and the focal adhesion pathway were also found to show changes in expression, which indicates that loss of DMP1-PG may also affect cell-cell interactions.

## Discussion

In bone matrix, DMP1 protein is present in four forms: a 57 kDa DMP1 C-terminal fragment, a 37 kDa DMP1 N-terminal core protein, a glycosylated N-terminal core protein (DMP1-PG) and trace amount of full-length DMP1. Among them, DMP1-PG is highly expressed in bone matrix. As an amino acid motif specialized for the attachment of GAG chains to serine, the Ser-Gly sequence (Ser^89^-Gly^90^ in the mouse amino acid sequence) of DMP1 is highly conserved among species^9^. It was also demonstrated that glycosylation of DMP1 could be fully blocked in HEK-293 cells by the S89G substitution^13^. Based on our *in vivo* experiments, the S89G substitution could reduce the glycosylation level of DMP1 in the bone matrix of S89G-DMP1 mouse long bones, which confirmed that Ser^89^ of mouse DMP1 is a key glycosylation site. These findings lead to the question of why the glycosylation of DMP1 is fully blocked by S89G *in vitro* but not *in vivo*. Also, it is important to determine whether there is a backup glycosylation site for mouse DMP1 *in vivo* or the blocking efficiency of S89G is not strong enough. Based on our data, it is still difficult to explain the differential blocking efficiency of S89G substitution between *in vitro*^13^ and *in vivo* conditions. Although S89G substitution could not fully block DMP1 glycosylation *in vivo*, we have shown that this reduction in bone mass was due to the loss of DMP1-PG, and the bone morphological phenotypes of S89G-DMP1 mice are similar to those found in mice lacking other well-known bone proteoglycans^4^.

It is also worth discussing why DMP1 N-terminal fragments exist as proteoglycans in bone. As one category of highly expressed proteoglycans in bone, DMP1-PG should have a unique biological function in osteogenesis. Using classical bone matrix protein extraction methods, DMP1-PG was found to be highly expressed in unmineralized bone matrix surfaces and in articular cartilage^11^,^12^. Further immunohistochemistry staining showed that DMP1 N-terminal staining was mainly observed either in newly formed osteoid (the mineralization front) surrounding osteocytes^11^ or in the matrix of articular cartilage (on the surface of joints)^12^. Based on our observations, the biological function of DMP1-PG could be partially explained by the bone phenotypic changes in S89G-DMP1 mice. In S89G-DMP1 mice, a decrease in the trabecular bone volume and the BV/TV ratio indicated that bone remodeling was inhibited; the decreased stiffness of cortical bone, combined with an increase in bone material density, indicated that the mineralization level of bone matrix was affected; other than that, the thinner articular cartilage and narrower osteoid layer that were observed in S89G-DMP1 mice indicated that pathological substitution of osteoid/cartilage with mineralized bone occurred at the mineralization front. All these phenotypes were related to a loss of proteoglycans after the reduction in DMP1-PG expression. As is well known, proteoglycans are important components of bone remodeling microenvironments for osteoblasts, and a loss of proteoglycans will lead to abnormalities in both bone mineralization and bone remodeling^2^. A key role for DMP1-PG in bone development was corroborated by the observation that loss of DMP1-PG leads to delayed osteogenesis in both epiphyseal and cortical bone areas. Specifically, mice deficient in DMP1-PG developed age-dependent osteopenia and had multiple metabolic defects in their osteoblasts, suggesting that DMP1 works in a similar way as Biglycan in modulating the activity of osteoblasts^14^. Additionally, along with the inhibition of DMP1 glycosylation, other bone matrix proteoglycans, such as biglycan and decorin, were also down-regulated. All of these PGs are important for regulating biomineralization. Thus, a loss of or decrease in DMP1-PG would inhibit the expression of other bone proteoglycans and ultimately disturb osteoid formation.

Interestingly, DMP1 is well known as a regulator of mineralization and serum phosphate resorption^5^,^6^, and the *Dmp1*-KO mouse is a typical rickets mouse model with osteomalacia phenotypes, which are attributed to the loss of functional DMP1 C-terminal fragments^8^. In *Dmp1*-KO long bones, the cell lacunae of osteocytes, along with the osteoid area, increased and the mineralized level decreased^6^. In contrast, the phenotypes of S89G-DMP1 mouse long bones were different from those of *Dmp1*-KO mice. After the S89G substitution, the DMP1-N terminal lost its proteoglycan characteristics, which led to the acceleration of mineralization surrounding the osteocytes. The size of osteocyte lacunae in S89G-DMP1 mice decreased as the osteoid thickness decreased. The differences between *Dmp1*-KO and S89G-DMP1 mice indicate that DMP1-PG (glycosylated DMP1 N-terminal) and the DMP1-C terminal have different roles *in vivo*: DMP1-PG is mainly expressed in unmineralized osteoid^11^ and may inhibit or regulate biomineralization, and the C-terminal of DMP1 (highly phosphorylated) is expressed in the mineralized area^11^ and may help accelerate matrix mineralization^8^,^15^.

Another novel finding in S89G-DMP1 mice is the abnormal phenotypes of osteocytes. According to the histology and TEM data and the related histological findings, the nucleo-cytoplasmic ratio of osteocytes increased in S89G-DMP1 cortical bone, especially in the area of the bone mineralization defect. Furthermore, the decreased space for intracytoplasmic organelles will affect the transcriptional and translational processing of bone formation genes. We assumed that loss of proteoglycans would disturb the rhythm of mineralization and lead to excessive mineralization of extracellular matrix. Because of the important role of the extracellular matrix environment in the activities of bone cells^16^,^17^, changes in bone mineralization and a harder bone matrix would stimulate osteocytes and affect the bioactivity of osteocytes. Down-regulation of proteoglycans and other osteogenic genes should be a result of osteocyte lesions^18^.

Finally, the differential expression analysis of mRNA extracted from bones of the two groups showed that several important osteogenesis pathways were affected after the inhibition of glycosylation of DMP1. Among them, the TGF-β and ECM-cell interaction pathways are directly related to cartilage and bone formation^19^,^20^. To date, key roles of two proteoglycans in controlling bone mass by regulating TGF-β activity have been uncovered^17^, which could help us find out how DMP1 works in the future. In addition, changes in molecules involved in pathways related to cell adhesion, tight junctions and focal adhesion indicate that the proteoglycan form of DMP1 may also mediate adhesion signaling in bone and cartilage formation^21^,^22^,^23^.

In summary, we first showed that proteoglycan forms of DMP1 could control bone mass and remodeling. Our findings shed light on: (1) functions of the highly expressed acidic proteoglycan DMP1-PG in regulating osteogenesis; (2) the importance of microenvironments created by proteoglycans in both bone development and the maintenance of osteoblast/osteoclast activity; and (3) the maintenance of proper mineralization and skeletal metabolism by proteoglycans. Further investigations of the S89G-DMP1 mutation model should focus on the following questions: will the loss of DMP1-PG delay fracture healing and generate weaker bones after fracture repair in S89G-DMP1 mice; could DMP1-PG be used therapeutically to enhance the healing of skeletal critical size defects by tissue engineering or systemic application; what is the role of DMP1-PG in cell interaction signaling; and what is the specific regulation mechanism of DMP1-PG for differentiation of osteoblasts, osteocytes, osteoclasts and BMSCs; is there a backup glycosylation site for S^89^ in mouse DMP1 *in vivo*? As a newly identified proteoglycan in bone development, the essential downstream pathways and underlying receptors of DMP1-PG also need to be identified. All these additional studies are required before we can understand the exact mechanisms of DMP1-PG in bone development.

## Methods

### Generation of DMP1 point mutation mice

A mouse model expressing only S89G-Dmp1 was created using a homologous recombination method by Beijing Biocytogen (Beijing, China). Briefly, the S89G mutation was introduced into exon 6 using an overlap extension-PCR method. Homology regions covering 6.6 kb upstream of DMP1 exon 6 and 8.2 kb downstream of exon 6 were subcloned from a BAC clone (RP23-314P12; Invitrogen, USA) from a C57BL/6J mouse genomic BAC library. An FRT-flanked Neo resistance positive selection cassette was inserted to 179 bp downstream of exon 6. The complete sequence of the targeting vector was verified by full sequencing. After linearization, the targeting vector was transfected into C57BL/6J embryonic stem (ES) cells (Biocytogen, Beijing, China) by electroporation. Eight positive clones were identified by Southern blotting with a 5′probe and a 3′probe. Three positive clones were injected into Balb/c blastocysts and implanted into pseudopregnant females. Five chimeric male mice were crossed with C57BL/6J females to obtain F1 mice carrying the recombined allele containing the S89G mutation and the Neo selection cassette. The resulting pups were studied for germinal line transmission of the recombination event using the PCR strategy. The presence of the S89G mutation was further verified by sequencing. Heterozygous males were mated with B6.129S4-Gt (ROSA) 26- Sortm1 (FLP1) Dym/RainJ females (Jackson Laboratories) to remove the NEO cassette. The elimination of the cassette in the offspring was verified by PCR with the primers Frt-F and Frt-R. Homozygous mutant mice were obtained by inter-crossing heterozygous littermates. Eight positive clones were selected, and 3 pups were collected from the first litter. Genotyping of S89G-DMP1 mouse-primers used for S89G point mutation identification included the following: F-primer, 5′-GCTCGACTAGAGCTTGCGGA-3′, and R-primer, 5′-CGTCTCACATGGTAGTGGAGA-3′. Primers for WT and S89G-DMP1 mouse are designed and used for distinguishing endogenous Dmp1 and S89G-Dmp1. Product size of endougenous DMP1 is 308 bp and S89G-DMP1 is 306 bp. Animals were raised in a specific pathogen-free (SPF) facility, under a 12:12 h day–night illumination cycle. For sacrificing mice, animals were killed by cervical dislocation after inhalation anesthesia. The animal protocol was approved by the Animal Welfare Committee of School of Stomatology, Tongji University (Shanghai, China). All experiments were performed in accordance with relevant guidelines and regulations.

### Extraction, separation and detection of DMP1

Noncollagenous proteins (NCPs) were extracted from long bones of four 2-month-old WT and S89G-Dmp1 mice, as previously described^24^. The extracts were loaded onto a 10 ml Q-Sepharose ion-exchange chromatography column (Amersham Biosciences, USA) to separate acidic proteins. The elution gradient employed was 0.1–0.8 M NaCl/6 M urea at pH 7.2. Acidic NCPs, including DMP1-PG and the core protein of DMP1-N terminal (37 kDa), were eluted into eight fractions. These fractions were analyzed by SDS-PAGE and Western immunoblotting to identify DMP1 and its processed fragments. Monoclonal anti-DMP1-N 9B6.3 antibody^9^ was used at a concentration of 3 μg/ml. Note that 9B6.3 antibody could identify three forms of DMP1: DMP1-PG (high molecular weight smear), N-terminal fragment core protein (about 37 kDa), and full-length form of DMP1. Alkaline phosphatase-conjugated anti-mouse IgG (Sigma, USA) at a dilution of 1:3000 was employed as the secondary antibody for the Western immunoblotting analysis. The blots were incubated in the chemiluminescent substrate ECL (Sigma, USA) for 5 min, and exposed under a chemiluminescent measurement machine.

### X-ray radiography and μ-CT analysis of long bone

Hind legs of S89G-DMP1 mice were compared to those of wild type mice. Under anesthesia, 2- and 6-month-old mice were perfused from the ascending aorta with 4% paraformaldehyde. The hind legs were dissected and further fixed in the same fixative for 48 h. The long bones of the 2- and 6-month-old mice were dissected. The density of long bones was compared by X-ray radiography (Faxitron, USA) and μCT-35 (Scanco Medical, Switzerland). The μ-CT analyses included a high-resolution scan of the whole femur (6-μm slice increment) and the femoral metaphysis region proximal to the distal growth plate for evaluation of trabecular bones. For trabecular bone analysis, we selected a cylinder area in the center of the metaphysis region with a radius of 100 μm and a length of 1200 μm (200 slices). Data acquired were used for quantitative analyses. The μ-CT parameters obtained and analyzed included bone volume to total volume ratio (BV/TV), apparent density, material density, trabecular number (Tb.N), trabecular thickness (Tb.Th), trabecular separation (Tb.Sp) and material density (Mat.Den). Four samples were tested in each group.

### Histology and morpholog

Following demineralization in 10% EDTA (pH 7.4) at 4 °C for four weeks, specimens were embedded in paraffin and cut into 5-μm-thick sections. Histological characterization of tissue from 2- and 6-month-old mice was performed by Hematoxylin and Eosin (H&E) staining. Additionally, DAPI staining was used for cell nucleus staining in bone matrix. Transmission electron microscopy (TEM) and scanning electron microscopy (SEM) were used for demonstration of morphological changes in osteocytes. Specimens of 2-month-old mice were fixed with 4% paraformaldehyde and 1% glutaraldehyde in 0.1 M sodium cacodylate buffer (pH 7.2). Samples were then fixed with 1% osmium tetroxide, and processed for embedding in white acrylic resin. Observations were conducted using an H-7650 transmission electron microscope (Hitachi, Japan). For describing the changes in the osteocyte lacunocanalicular system, subtle changes in osteocytes were detected by SEM. Bone tissues were fixed in 70% ethanol and coated with gold and palladium. Histological changes in osteocytes were examined by a PHILIP QUANTA-200 SEM. Long bone samples of *Dmp1-*KO mice was a gift from Dr. Jian Q. Feng’s lab in the Baylor College of Dentistry.

### RT-qPCR test for RNA from bone cells

To assess the expression levels of osteogenesis-related markers, real-time quantification PCR (RT-qPCR) was used. Total RNA from cortical bone of 4-month-old femurs was extracted using Trizol®reagent (Invitrogen, USA). First-strand cDNA was synthesized from 1 μg of total RNA using a Transcriptor First Strand cDNA Synthesis Kit (Roche, Swiss) in a final volume of 20 μl with oligoDT primers. Expression of target genes and internal reference genes was evaluated by quantitative real-time PCR with a Faststart Essential DNA Green Master Kit (Roche, Swiss). The primers were designed and synthesized (Invitrogen, China). Primers are listed in Table 2. All reactions were performed in a total volume of 20 μl and contained 50 ng of reverse transcribed RNA using a LightCycler 96 Instrument system (Roche, USA). The thermo-cycler conditions were preincubation at 95 °C for 600 s, 45 cycles of 95 °C for 10 s, 60 °C for 10 s, 72 °C for 20 s, melting at 95 °C for 10 sec, 65 °C for 60 s and 97 °C for 1 s. Three Samples were collected in each group, and reactions were run in triplicate. The mouse GAPDH gene was used as an internal control for each sample. The relative mRNA expression levels of the genes were calculated using the 2^–△△Ct^ calculation method.

### ECM protein expression levels

For examining the expression and immunolocalization of major ECM proteins in long bones, anti-DMP1-N-9b6.3 monoclonal antibody, anti-DMP1-C-8G10.3^12^ monoclonal antibody, anti-BSP-10D9.2^12^ and anti-OPN monoclonal antibody^12^ (Santa Cruz, USA) were used at a dilution of 1:400. All immunohistochemistry experiments were performed using the ABC kit and DAB kit (Vector Laboratories, USA). Additionally, the same antibodies were used for the detection of BSP and OPN by Western immunoblotting.

### Osteoblast activity *in vitro*

Five three-day-old WT or DMP1-S89G mice were sacrificed. The skin and brain were carefully removed from the skull. The calvarias were gently washed in PBS and incubated in 1% trypsin for 5 min and 0.2% collagenase solution for 30 min at 37 °C. After removing the collagenase, the cells were harvested by centrifugation at 1,500 g for 5 min. Cells were cultivated in 24-well plates with α-MEM culture medium/10% FBS until the cells reached confluence. *In vitro* osteoblastic differentiation tests were performed using a P3 generation of osteoblasts in medium containing 50 μg/ml of ascorbic acid, 10 nM dexamethasone and 5 mM β-glycerophosphate (Sigma, USA). The medium was changed every 3 days. After 21 days of induction, the cells were fixed in 4% PFA for 1 h and rinsed with distilled H_2_O. Cells were stained with 40 mM Alizarin Red solution (Sigma, USA) for 15 min and then rinsed with distilled H_2_O five times and PBS for 15 min. The mineralized nodule areas were quantified using Nikon image software and expressed as a percentage of Alizarin Red-positive area per total area. Images were taken using a Nikon Ti inverted microscope (Nikon, Japan).

### Osteoclast activity *in vivo*

Trap staining was used to examine the bone resorption activities and the activities of osteoclasts in the bone remodeling area. In 6-month-old mice, the osteoclast number per view and the osteoclast surface per bone surface were calculated and compared.

### Bone Mechanical property testing

The bone strength of the femur and tibia diaphysis of 3-month-old mice were evaluated using three-point bending tests that were conducted with an FR-108B testing machine (Farui Co., China). The crosshead speed in the test was 10 mm/min. The ultimate bending load (N) and stiffness (N/mm) were determined from the load–displacement curve. The greatest displacement and the maximum force during the three point tests were determined.

### RNA sequencing and functional enrichment analysis of S89G-DMP1 mouse long bones

Sequencing was performed at Guangzhou Ribo Bio Co., Ltd. with the Illumina HiSeq 2500. Total RNA was isolated from long bones of WT or S89G-DMP1 mice using Trizol (Invitrogen, USA) according to the manufacturer’s protocol. RNA purity was assessed using a NanoVue (GE, USA). Each RNA sample had an A260:A280 ratio greater than 1.8 and an A260:A230 ratio greater than 2.0. RNA integrity was evaluated using the Agilent 2200 Tape Station (Agilent Technologies, USA) and each sample had an RIN above 7.0. Briefly, mRNAs were isolated from the total RNA and fragmented to approximately 200 bp. Subsequently, the collected mRNAs were subjected to first strand and second strand cDNA synthesis followed by adaptor ligation and enrichment with a low-cycle according to the instructions provided with the TruSeq^®^ RNA LT/HT Sample Prep Kit (Illumina, USA). The purified library products were evaluated using the Agilent 2200 TapeStation and Qubit^®^2.0 (Life Technologies, USA) and then diluted to 10 pM for cluster generation *in situ* on the HiSeq2500 pair-end flow cell followed by sequencing (2 × 100 bp) using a HiSeq 2500. The sequenced raw data were filtered to remove low-quality tags, empty reads, and reads with only one copy number. Tophat was used to align the remaining clean reads to the sequences in a mouse genome database, allowing up to two base mismatches. The mapped clean reads were designated as unambiguous clean reads. For two-factor analysis of variance, the number of unambiguous clean reads for each gene was calculated and normalized to log-counts per million using the latest version of the limma package. All the differentially expressed genes were used for heat map analysis and KEGG ontology enrichment analyses. For KEGG enrichment analysis, a Q-value <0.05 was used as the threshold to determine significant enrichment of the gene sets.

### Statistical analysis

Data analysis was performed using Student’s t tests for two-group comparison. Results are represented as the mean ± standard error (SEM). p < 0.05 was considered statistically significant.

## Additional Information

**How to cite this article**: Sun, Y. *et al.* Glycosylation of Dentin Matrix Protein 1 is critical for osteogenesis. *Sci. Rep.* **5**, 17518; doi: 10.1038/srep17518 (2015).

## Supplementary Material

Supplementary Information

## Figures and Tables

**Table 1:** Kegg analysis for the differential expression of genes in WT and S89G-DMP1 mice.

Gene term	count
Porphyrin and chlorophyll metabolism	12
Leukocyte transendothelial migration	20
Glycolysis / Gluconeogenesis	15
ECM-receptor interaction	15
Glutathione metabolism	11
Focal adhesion	21
Viral myocarditis	12
Hematopoietic cell lineage	11
Complement and coagulation cascades	10
Tight junction	13
Cell adhesion molecules (CAMs)	14
B cell receptor signaling pathway	9
Pentose phosphate pathway	5
Fc gamma R-mediated phagocytosis	10
Systemic lupus erythematosus	10
Glycerolipid metabolism	6
Type I diabetes mellitus	7
Nitrogen metabolism	4
TGF-beta signaling pathway	8
Pyruvate metabolism	5
Allograft rejection	6
Graft-versus-host disease	6

*mRNA used in this analysis is from long bone.

**Table 2:** Primers used in this study.

Gene	F/R	Sequence
DMP1-Neo-F	forward	GTGCCATTGAAGCATTACCTCATG
DMP1-WT-R	reverse	GGTTCTTACATGGGCAGGATAAGC
DMP1-WT-F	forward	5′-CGCATTGTCTGAGTAGGTGTC-3′
DMP1-WT-R	reverse	5′-GGTTCTTACATGGGCAGGATAAGC-3′
Runx2	forward	5′-GCACAAACATGGCCAGATTCA-3′
Runx2	reverse	5′-AAGCCATGGTGCCCGTTAG-3′
Ocn	forward	5′-AGGGAGGATCAAGTCCCG-3′
Ocn	reverse	5′ -GAACAGACTCCGGCGCTA-3′
Osx	forward	5′-AGAGGTTCACTCGCTCTGACGA-3′
Osx	reverse	5′-TTGCTCAAGTGGTCGCTTCTG-3′
Rankl	forward	5′-CACACCTCACCATCAATGCT GC-3′,
Rankl	reverse	5′-GAAGGGTTGGACACCTGAA TGC-3′
Col-1	forward	5′-GGTCCTCGTGGTGCTGCT-3′
Col-1	reverse	5′-ACCTTTGCCCCCTTCTTTG-3′
Acan	forward	5′-CCATCTCCTCAGCGAAGCAG-3′
Acan	reverse	5′-CTACAAGGACAGTGACTTTG-3′
Dcn	forward	5′-CGAGTGGTCCAGTGTTCTGA-3′
Dcn	reverse	5′-AAAGCCCCATTTTCAATTCC-3′
Bgn	forward	5′-GGCCTCCAGCACCTCTACGCC-3′
Bgn	reverse	5′-AACACGCCCTTGGGCACTTT-3′
Opn	forward	5′-GATCAGGACAACAACGGAAAGG-3′
Opn	reverse	5′-GCTGGCTTTGGAACTTGCTT-3′
Bsp	forward	5′-AGGACTGCCGAAAGGAAGGTTA-3′
Bsp	reverse	5′-AGTAGCGTGGCCGGTACTTAAA-3′
Dmp1	forward	5′-AGTGAGTCATCAGAAGAAAGTCAAGC-3′
Dmp1	reverse	5′-CTATACTGGCCTCTGTCGTAGCC-3′
Alp	forward	5′-GCCCTCTCCAAGACATATA -3′
Alp	reverse	5′-CCATGATCACGTCGATATCC-3′
DKK1	forward	5′-TCCCCTGTGATTGCAGTAAA-3′;
DKK1	reverse	5′-TCCAAGAGATCCTTGCGTTC-3′;
