# Supplementary material for: Glycosylation of Dentin Matrix Protein 1 is critical for osteogenesis
Source: Sci Rep. 2015 Dec 4;5:17518. doi: 10.1038/srep17518 (PMC4669440; doi:10.1038/srep17518)
Supplement: Supplementary Information [file srep17518-s1.pdf]

Supplementary Material

Glycosylation of Dentin Matrix Protein 1 is critical for osteogenesis

Yao Sun[1,2,+], Yuteng Weng[1,+], Chenyang Zhang[1], Yi Liu[1], Chen Kang[2], Zhongshuang Liu[1], Bo Jing[3], Qi Zhang[1], Zuolin Wang[1,*]

1, Tongji University, School of Stomatology, Laboratory of oral biomedical science and translational medicine, Shanghai, China, 200072

2, Harbin Medical University, Institute of Hard Tissue Development and Regeneration, Harbin, China, 150086

3, Tongji University, School of medicine, stem cell center, Shanghai, China, 200072

***Correspondence and requests for materials should be addressed to:** Zuolin Wang, email: zuolintongji@126.com

+These authors contributed equally to this work.

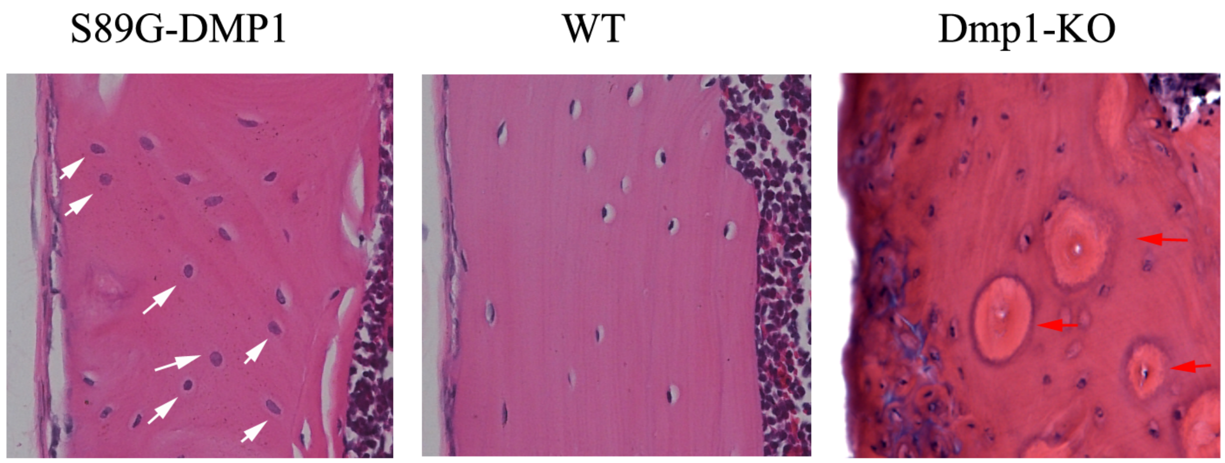

Fig.S1 H&E staining for long bone of WT, Dmp1-KO and S89G-DMP1 mouse.

Compared with WT mouse, the osteocytes of S89G-DMP1 had enlarged cell nucleus and lost cell lacunae space (white arrows); the *Dmp1*-KO mouse long bone contains more osteoid areas (red arrows) than WT mouse.
